# Alcohol intake and cardiovascular risk factors: A Mendelian randomisation study

**DOI:** 10.1038/srep18422

**Published:** 2015-12-21

**Authors:** Yoonsu Cho, So-Youn Shin, Sungho Won, Caroline L Relton, George Davey Smith, Min-Jeong Shin

**Affiliations:** 1 Department of Public Health Sciences, BK21PLUS Program in Embodiment: Health-Society Interaction, Graduate School, Korea University, Seoul, Republic of Korea; 2 MRC Integrative Epidemiology Unit, School of Social & Community Medicine, University of Bristol, Bristol, UK; 3 Department of Public Health Science, Seoul National University, Seoul, Republic of Korea; 4 Institute of Genetic Medicine, Newcastle University, Newcastle upon Tyne, UK.; 5 Korea University Guro Hospital, Korea University, Seoul, Republic of Korea

## Abstract

Mendelian randomisation studies from Asia suggest detrimental influences of alcohol on cardiovascular risk factors, but such associations are observed mainly in men. The absence of associations of genetic variants (e.g. rs671 in *ALDH2*) with such risk factors in women – who drank little in these populations – provides evidence that the observations are not due to genetic pleiotropy. Here, we present a Mendelian randomisation study in a South Korean population (3,365 men and 3,787 women) that 1) provides robust evidence that alcohol consumption adversely affects several cardiovascular disease risk factors, including blood pressure, waist to hip ratio, fasting blood glucose and triglyceride levels. Alcohol also increases HDL cholesterol and lowers LDL cholesterol. Our study also 2) replicates sex differences in associations which suggests pleiotropy does not underlie the associations, 3) provides further evidence that association is not due to pleiotropy by showing null effects in male non-drinkers, and 4) illustrates a way to measure population-level association where alcohol intake is stratified by sex. In conclusion, population-level instrumental variable estimation (utilizing interaction of rs671 in *ALDH2* and sex as an instrument) strengthens causal inference regarding the largely adverse influence of alcohol intake on cardiovascular health in an Asian population.

Previous epidemiological studies have reported potential beneficial effects of moderate alcohol intake on cardiovascular health^1^,^2^. In a recent review paper combining results from 84 observational studies, moderate drinkers were shown to have reduced risks of cardiovascular disease outcomes compared with non-drinkers, although heavy drinkers had the highest risks of all^3^. However, such evidence is not adequate for promotion of moderate alcohol use in prevention of heart disease given the known limitations of observational studies^2^,^4^. First, observed cardio-protective effects may be a form of reverse causation whereby individuals with the early stages of disease reduce their alcohol intake^2^,^4^,^5^. Second, observed effects might be due to confounding factors such as socioeconomic position, diet or other health-related behaviours and therapeutic regimes^4^. Therefore, the causal nature of association beyond observed correlation must be investigated in order to fully evaluate the benefits or harms of alcohol use.

Potential relationships should be interrogated through methods that can manipulate exposure and observe corresponding outcomes while accounting for confounding factors^6^. The gold standard is a randomised controlled trial (RCT), but this may be impossible to implement, prohibitively expensive or unethical. One alternative approach is that of Mendelian randomisation^7^. This method utilises a genetic variant that is allocated at conception in a manner that is independent of environment; people with the same genotype are thus akin to randomly allocated group of people in an RCT^8^. In essence, Mendelian randomisation exploits the idea such that a genetic variant, which proxies for the exposure, is expected to be related to the outcome to the degree anticipated given its association with the exposure. When Mendelian randomisation is implemented as a form of instrumental variable analysis, the genetic variant is referred to as an instrumental variable (IV)^9^. Using a Mendelian randomisation approach, causal effects of alcohol intake on cardiovascular outcomes have been investigated in several studies^5^,^10^,^11^,^12^. Robust IVs for alcohol intake include genetic variants in aldehyde dehydrogenase 2 (*ALDH2*)^13^,^14^ and alcohol dehydrogenase 1B (*ADH1B*)^10^. Both these genes are involved in alcohol metabolism (Supplementary Fig. S1), although the *ALDH2* variants have substantially more influence on alcohol intake than the *ADH1B* variants^10^. The *ALDH2* variants are polymorphic mainly in East Asian populations (Supplementary Fig. S2). Individuals who carry the variant allele experience on average greater discomfort after drinking alcohol, including nausea and facial flushing (so called Asian flush) since the variant allele codes for an inactive form of the enzyme, that leads to build-up of acetaldehyde in the circulation following alcohol consumption. As a result, carriage of the *ALDH2* variant has consistently been linked with drinking behaviours^15^,^16^,^17^,^18^,^19^ and alcohol related diseases or risk factors in a number of Asian population studies^11^,^15^,^20^,^21^,^22^,^23^,^24^,^25^,^26^. For example, these studies consistently suggest that alcohol intake is associated with higher blood pressure^21^,^24^, not only in heavy drinkers but also in moderate drinkers^11^, corroborating some observational epidemiological studies^3^. Use of the variant also implies alcohol drinking is associated with coronary artery disease^25^ and coronary spastic angina^26^. On the other hand, some studies suggest alcohol drinking may have a favourable influence by increasing high density lipoprotein (HDL) cholesterol or decreasing low density lipoprotein (LDL) cholesterol^11^,^22^. However, these findings are not always robust^10^,^25^ and furthermore, the causal relationship between HDL cholesterol and cardiovascular health is uncertain^27^,^28^.

For cardiovascular outcomes showing association with the *ALDH2* variant, associations have largely been confined to Asian men^5^,^21^,^24^. Weak or null associations observed in Asian women are due to a low level of alcohol consumption in females irrespective of the genotype, which is analogous to the situation within a RCT framework where randomly allocated groups receiving a very low amount of exposure would not result in any difference in outcomes^21^. The difference of associations between men and women provides an excellent rationale that the variant influences outcomes only through the exposure (i.e. alcohol intake), validating an assumption of Mendelian randomisation. The reasoning behind this is that if it were not the case – for example, if pleiotropic effects of the genetic variant influenced the outcomes - the same association between the variant and outcomes would have been seen in women as well as in men, as discussed in detail elsewhere^5^,^20^. Nevertheless, this sex stratification of alcohol intake also raises a question whether using the rs671 genotype alone as an IV would be sufficient to properly assesses causal effects in the whole population when both genetic variants and sex influences on alcohol intake should be considered^29^.

In this study, we carried out a Mendelian randomisation study to investigate the causal effects of alcohol intake on a range of cardiovascular outcomes and included the stratification of alcohol intake by gender. Data were collected from a total of 7,152 individuals from South Korea, including 3,365 men and 3,787 women. First, causal effects of alcohol intake were investigated in men and women separately, by conventional IV models using the rs671 genotype in *ALDH2* as an IV. To demonstrate that the observed sex differences of association between the variant and cardiovascular outcomes were due to difference in corresponding drinking level rather than some particular influence of sex, the male-specific association was subsequently evaluated in sub-groups of never-drinkers and ever-drinkers. Finally, population-level causal effects were estimated by an extended IV model utilising interaction of the rs671 genotype and sex as an IV.

## Results

### General characteristics

Basic characteristics of male and female participants are shown in Table 1. Mean values were different between men and women in most variables with the exception of hip circumference, total cholesterol, the rs671 genotype and genotypic principal components. Men were younger, more likely to live in urban areas, more educated, doing less exercise and smoking more than women on average. Alcohol intake was considerably higher in men than women; 72% of men were current drinkers compared to 26% of women; the average alcohol intake was 18.8 ± 0.5 (g/day) in men and 1.3 ± 0.1 (g/day) in women; and gamma- glutamyl transpeptidase (GGT) was 55.4 ± 1.6 (IU/L) in men and 19.0 ± 0.3 (IU/L) in women. Men had higher prevalence of diseases and more generally unfavourable risk factors than women, although men had a few more generally favourable values as well (lower body mass index (BMI) and LDL cholesterol). It should be noted that there was no difference between men and women in the prevalence of rs671 genotype and genotypic principal components.

Characteristics were also provided according to the rs671 genotype in each sex group (Table 2). In both men and women, the rs671 genotype was in Hardy-Weinberg equilibrium with the A-allele frequency of 16%. The rs671 genotype was not associated with lifestyle or socioeconomic factors. Regarding alcohol intake, carriers of the rs671 A-allele had a lower proportion of current drinkers and consumed less alcohol than non-carriers in both men and women, though the magnitude of difference was bigger in men. With regard to disease prevalence and related risk factors, carriers of the rs671 A-allele appeared to have several potentially beneficial effects and a few potentially adverse effects than non-carriers (Table 2). All these associations were observed only in men and not in women. In addition to lifestyle or disease related factors, potential population stratification of the rs671 genotype was investigated through its association against the first five genotypic principal components. The second and the fourth principal components were correlated with the rs671 genotype in men (p = 0.02) and women (p = 0.04), respectively. Hence, these two principal components were included in the subsequent Mendelian randomisation analysis to correct for population stratification.

Next, the male population was divided into two groups by their drinking status: ever-drinkers and never-drinkers, and then corresponding characteristics in each group were provided according to the rs671 genotype (Table 3). In this stratified analysis, potential collider bias^30^ was tested using generalized regression models with an interaction of genotype and drinking behaviour. Strong evidence of collider bias was observed for smoking behaviour (interaction p < 0.0001) where its association with genotype by strata were in opposite directions (Table 3). To minimize the effect of risk factors susceptible to collider bias, associations between the rs671 genotypes and cardiovascular outcomes were then assessed with adjustments for smoking with the results being closely similar to those without adjustments. In male ever-drinkers, the rs671 A-allele was associated with several potentially beneficial effects and a few potentially adverse effects after adjustments (Table 3). These associations were not observed in male never-drinkers, apart from weak associations with waist to hip ratio and fasting glucose level.

### Observational associations

Association results based on the ordinary least squares (OLS) regression models can be found in Table 4 and Supplementary Tables S1. In men, alcohol intake was shown to be associated with higher hypertension risks, blood pressure, BMI, waist circumference, waist to hip ratio, log-transformed fasting blood glucose, HDL cholesterol, log-transformed triglycerides as well as with lower LDL cholesterol. In women, alcohol intake was associated with higher hypertension risks, blood pressure, BMI, hip circumference, log-transformed fasting glucose, total cholesterol and HDL cholesterol. The heterogeneity of OLS estimates in men and women was observed for diastolic blood pressure, log-transformed fasting blood glucose and HDL cholesterol and marginally for hypertension, hip circumference and total cholesterol under the fixed effect model. All regression analysis results were inspected based on plots of the dependent variable against the independent variable as well as plots of residuals against fitted values. Neither nonlinear association (such as U shape association) nor a structured pattern of residual distribution was evident (data available on request).

### Causal estimates from Mendelian randomisation analysis

Causal effects of alcohol intake on cardiovascular health and life style factors were inferred by IV estimation techniques (Table 5 and Supplementary Table S2). Corresponding causal relationships were also assessed through the association of the rs671 genotype with cardiovascular health and life style factors (Supplementary Tables S3 and S4). In men, alcohol intake, instrumented by the rs671 genotype, was associated with higher risks of hypertension, blood pressure, waist circumference, waist to hip ratio, log-transformed fasting blood glucose, HDL cholesterol, and log-transformed triglycerides as well as with lower LDL cholesterol (all p < 0.05). In women, there was little evidence for causal influences of alcohol intake on cardiovascular outcomes with an exception of hip circumference (p = 0.035). The heterogeneity of IV estimates in men and women was observed for hip circumference (p = 0.038) under the fixed effect model.

Population-level causal effects were assessed as IV estimates where interaction of the rs671 genotype and sex was used as an IV given that alcohol intake was stratified by sex as well in the whole population (Table 6). As a result, one unit of alcohol intake (g/day) was associated with higher hypertension risks, blood pressure, waist to hip ratio, log-transformed fasting blood glucose, HDL cholesterol, log-transformed triglycerides as well as with lower LDL cholesterol at a population level.

## Discussion

Here, we present a Mendelian randomisation study on alcohol intake and cardiovascular outcomes by analysing 7,152 individuals (3,365 men and 3,787 women) in South Korea. Causal influences of the exposure cannot be properly measured if the exposure level (alcohol intake, in this study) is indistinguishably low although there is a potentially valid IV (the rs671 genotype, in this study). For this reason, potential health outcomes consequent on alcohol drinking are not easily assessed in Asian women compared to Asian men^5^,^21^. We first replicated null or weakly observed association of the rs671 genotype and cardiovascular outcomes in women. Furthermore, we ensured such null association in women was not because of any female-specific biological mechanism but because of low drinking levels, by demonstrating analogous null association in male never-drinkers. The average alcohol intake level was 18.8 g/day in men and 1.3 g/day in women, and 22.9 g/day and 0.0 g/day in male ever- and never-drinkers.

We quantified influences of alcohol intake on a wide range of cardiovascular outcomes by using instrumental variable estimation techniques. In men, one unit of alcohol intake (g/day), explained by the rs671 genotype, was associated with higher hypertension risks, and higher level of systolic blood pressure, diastolic blood pressure, waist circumference, fasting blood glucose, HDL cholesterol, triglycerides, and with lower LDL cholesterol. In women, none of these associations were observed as expected due to a very low alcohol intake. In the whole population, alcohol intake instrumented by interaction of the rs671 genotype and sex, appeared to have the same effects on cardiovascular outcomes as in the male population, although the confidence intervals of the effect sizes were larger.

Overall, we showed that alcohol intake is detrimental to most cardiovascular outcomes in the general Asian population as shown previously in Asian male populations^11^,^31^. The exception is high HDL cholesterol and low LDL cholesterol as they are generally considered favourable risk profiles with respect to cardiovascular health, although the protective role of high HDL cholesterol may not be fully established compared to that of low LDL cholesterol which has been supported by a number of Mendelian randomisation and RCT studies^1^,^27^,^28^,^32^.

The credibility of the rs671 genotype in *ALDH2* as an IV for alcohol intake has been discussed in many studies^11^,^12^,^14^,^20^,^21^. Biochemically, *ALDH2* encodes the main enzyme in alcohol metabolism transferring toxic acetaldehyde, into non-toxic acetate. Simultaneously, it prevents another toxic chemical, aldehyde, from accumulating in the body. People carrying a mutated allele of this gene that produces an inactive form of the ALDH2 enzyme (which is the case mainly in Asians), experience discomfort after drinking such as facial flushing, nausea and a rapid heartbeat. This is likely to be underlying reason for the association between *ALDH2* genotype and drinking behaviour.

In our data, supporting evidence was found that the rs671 genotype in *ALDH2* satisfied three core assumptions for an IV. First, it was independent of known confounders including age, education, residential area, physical activities and smoking status, both in men and women, as expected. Potential residual confounding by population stratification might exist to some extent, but we adjusted for genotypic principal components as covariates in the instrumental variable model. Second, it was strongly associated with alcohol intake (g/day) (F-statistic** = **262 in men and 38 even in women) confirming that it was an adequate IV unlikely to suffer weak instrument bias in this study. Furthermore, the rs671 genotype was also associated with other directly relevant alcohol-related traits. That is, people with slow alcohol metabolism due to carriage of a mutated allele (rs671 A-allele) appeared to have lower proportion of ever and current drinkers as well as lower levels of GGT. GGT is often used as a biomarker for heavy drinking and the lower levels of GGT are likely to be influenced by drinking less alcohol as suggested in the latest study^33^. The third assumption required for instrumental variable analysis (that the *ALDH2* genotype influences cardiovascular outcomes only through alcohol intake, in this study) is (like the assumption of no unmeasured confounding) impossible to validate. However, in this study, null effects in women provided evidence that it was unlikely that the estimated causal effects were due to pleiotropic effects; if there were pleiotropic effects, causal effects would have been observed in women as well as in men, as argued in detail elsewhere^5^,^21^.

Several studies have previously reported the causal relationship between alcohol intake and cardiovascular outcomes^10^,^11^,^20^,^21^,^22^,^23^. One of main strengths of the current study lies in relatively accurate estimation of population-level causal effects of alcohol intake when the alcohol intake is stratified by gender. Instead of the genotype alone, we formally used interaction of the genotype and sex as an IV for alcohol for the first time to our knowledge. Secondly, we considered a broad spectrum of cardiovascular outcomes compared to previously studies in an Asian population. For example, Chen *et al.* reported a sex-specific causal effect of alcohol intake on systolic and diastolic blood pressures and hypertension^21^ in their meta-analysis based on results extracted from published data, whereas we estimated both sex-specific and population-level causal effects on 12 additional outcomes in as large as or larger samples in individual level data. In another previous study, Kato *et al.* reported a strong sex-specific association of the rs671 genotype and blood pressures^22^ and also showed that such association was mediated by alcohol intake implying a causal relationship between alcohol intake and blood pressures, but their approach was limited in terms of quantification of the causal effects compared to the IV analysis that we applied in the current study. Finally it should be also mentioned that in a recent paper, Holmes *et al.* extensively covered the causal relationship of alcohol intake with various cardiovascular events and risk factors in the largest samples to date^10^; however, they used a different IV, the rs1229984 genotype in *ADH1B* which is known to be a much weaker IV than the rs671 genotype in *ALDH2* we used, as the latter is not polymorphic in European individuals of their study. Therefore, our current study is one of the most comprehensive study providing robust causal effects of alcohol intake on cardiovascular health outcomes. Another interesting feature of this study may be that it is one of the first Mendelian randomisation studies quantifying causal effects in alcohol intake in the Korean population, although there exists a relevant observational study^34^. The Korean population was selected, not only because it is an Asian population carrying a mutated rs671-A allele in *ALDH2*, but also because the population level alcohol intake is 71% and 84% higher than Japan and China, respectively, ranking it as the country with the highest level of heavy drinking in Asia (based on the alcohol per capita consumption on average between 2008 and 2010 in the 2014 WHO report). Our results were, however, consistent with those in other instrumental variable based studies in Japan^21^ and China^11^.

Nevertheless, our study has limitations. One of main limitation is the use of imputed genotype *ALDH2* rs671 although the imputed genotype was generated by a standardised protocol. Genotypes were quality controlled prior to imputation (based on missing call rates, minor allele frequency, Hardy-Weinberg equilibrium and sex match) and publicly available reference datasets were used with commonly used and previously evaluated software^35^,^36^. In addition, imputed genotypes were evaluated based on imputation quality score and Hardy-Weinberg equilibrium test. Thus, our imputed genotype would be as informative as a directly measured genotype, as shown in numerous genome-wide association studies. Also we acknowledge the limitation of the stratified analysis, as the stratification of the male population on drinking behaviour history (ever vs. never drinkers) could introduce collider bias^30^. We identified a risk factor susceptible to collider bias in our data, and adjusted for its effect on associations between genotype and cardiovascular outcomes, which produced little change in the effect estimates.

Despite providing evidence for a causal link between alcohol intake and a range of cardiovascular traits, our study did not observe clear causal effects of alcohol intake on cardiovascular disease or body mass index. This is consistent with previous evidence, such as that provided by Au Yeung *et al.* who reported a null effect of alcohol intake on cardiovascular disease in Chinese men^11^, although a recent meta-analysis by Holmes *et al.*^10^ reported strong effects on both in individuals of European descent. One possible explanation is that our study and the study by Au Yeung *et al.*^11^ were underpowered to detect the causal effect as the samples sizes were much smaller than those accrued by Holmes *et al.* (7,152, and 4,500 compared with 260,000, respectively)^10^. However, it is not straightforward to draw such conclusion yet because these studies have not only different sample sizes, but also different instruments (*ALDH2* genotype being a stronger instrument than *ADH1B* genotype), different ethnic backgrounds (Korean and Chinese compared with European) and different methods were used to define cardiovascular disease (self- report and self- report compared with combination of self- report, medical records, clinical/lab measures, death certificate and ICD code). Thus, a carefully designed large-scale study in well-phenotyped Asian population would be needed to further investigate this discrepancy.

In conclusion, this study indicates that a reduction in alcohol intake may be beneficial to cardiovascular health through avoiding detrimental influences on cardiovascular risk factors.

## Methods

### Study participants

Subjects for the analysis were obtained from two population based studies within the Korean Genome and Epidemiology Study (KoGES), the rural Ansung and urban Ansan cohorts. Detailed information for each study has been described elsewhere^37^. Briefly, the Ansung-Ansan cohorts were designed as longitudinal prospective studies initiated in 2001 and adopted the same investigational method. Participants in each cohort (5,018 in Ansung and 5,020 in Ansan aged 39–70) were recruited using a two-stage cluster sampling method. All participants took part in a health examination, interviews, and laboratory tests. The current study was based on the baseline data collected in 2001 from a total of 7,152 participants having the rs671 genotype in *ALDH2* available. All participants provided informed consent which was approved by the Human Subjects Review Committee at the Korea University Ansan Hospital or the Ajou University Medical Centre. The current study was approved by the Institute Review Board at the Korea University (KU-IRB-14-EX-153-A-1).

### Basic characteristics

Information was collected on demographic characteristics including age, area, education, physical activity and current smoking status. Education level was divided into four groups: elementary school, middle school, high school, or university. Physical activity was divided into two groups: practice or do not practice, according to whether or not the individual participated in any of the following daily activity types; intense physical activity at least 20 minutes, moderate physical activity at least 30 minutes, or walking at least 30 minutes. Current smokers were defined as a person who smoked cigarettes regularly at the time of the survey.

### Alcohol traits

Participants were also asked about their lifetime drinking behaviour, current drinking behaviour and detailed drinking behaviour over the previous 30 days, including frequency, amount and type of alcoholic beverages. Using this information along with average alcohol content of each beverage, total alcohol intake (g/day) was calculated. Further information on alcohol intake can be found in a previous publication^38^. As well as total alcohol intake (g/day), current drinking status, alcohol intake in current drinkers and GGT were considered as alcohol-related traits in this study. A current drinker was defined as an individual who drank alcoholic beverages regularly at the time of the survey. GGT concentration (IU/L) was measured from blood samples in the Seoul Clinical Laboratories (Seoul, Republic of Korea) collected after at least 8 hours of fasting.

### Blood pressure and other risk factors

Blood pressure was measured in a sitting position with a mercury sphygmomanometers after at least 5 minutes of rest. Two acceptable measurements of blood pressure were obtained within a 1 minute interval and recorded to the nearest 2 mmHg. Average measurements for systolic and diastolic blood pressure were used for statistical analysis. Height (cm) and body weight (kg) were measured to the nearest 0.1 cm or 0.1 kg without shoes, from which BMI (kg/m^2^) was derived. Waist circumference (cm) was measured at the narrowest part between the lower rib and the iliac crest to the nearest 0.1 cm, and the average of 3 repeated measurements was calculated. Hip circumference was measured at the widest portion of the buttocks to the nearest 0.1 cm, and the average of 3 repeated measurements was calculated. Waist to hip ratio was derived from waist circumference and hip circumference.

For laboratory tests, all participants had at least an 8 hour fasting period before blood collection. Collected blood samples were analysed in the Seoul Clinical Laboratories (Seoul, Republic of Korea) for assays including fasting blood glucose (mg/dL), total cholesterol (mg/dL), HDL cholesterol (mg/dL), and triglycerides (mg/dL). LDL cholesterol (mg/dL) was derived using the Friedewald formula^39^ in subjects with triglycerides less than 400 mg/dL as follows; LDL cholesterol = total cholesterol– HDL cholesterol– (triglycerides/5.0). For subjects with triglycerides of 400 mg/dL or more, LDL cholesterol value was marked as missing. GGT concentration (IU/L) was measured from the same blood samples.

### Disease outcome

According to health interview and examination, participants with self-reported diagnosed hypertension, use of blood pressure medicine, or measured systolic blood pressure greater than 140 mmHg, or diastolic blood pressure greater than 90 mmHg were considered as hypertensive. Cardiovascular disease status was defined by doctor-diagnosed and self-reported questionnaire information on myocardial infarction, congestive heart failure, coronary artery disease, peripheral blood vessel disease, and cerebrovascular disease. A coronary heart disease event was additionally defined by the same questionnaire information but only on myocardial infarction and coronary artery disease. Diabetes was defined by doctor-diagnosed and self-reported questionnaire information.

### Genotyping quality control and imputation

Detailed information is provided elsewhere^37^. Briefly, DNA samples were isolated from the peripheral blood of participants and genotyped using the Affymetrix Genome-Wide Human SNP array 5.0 (Affymetrix, Inc., Santa Clara, CA, USA). The accuracy of the genotyping was calculated by Bayesian Robust Linear Modelling using the Mahalnobis Distance genotyping algorithm^40^. A total of 352,228 SNPs in 8,842 participants became available after pre-imputation QC, 1) excluding SNPs with high missing genotype call rates (> 5%), with minor allele frequency (MAF) < 0.01, and not in Hardy-Weinberg equilibrium (HWE, P value < 1 × 10^−6^) and 2) removing samples with sex mismatch. Genetic principal components were computed in a subset of 304,225 SNPs after excluding additional 48,003 SNPs (not in HWE under a more conservative criterion, P value < 1 × 10^−5^) through the EIGENSTRAT software package^41^.

To impute rs671 *ALDH2* genotype (http://www.ncbi.nlm.nih.gov/projects/SNP/), all genotypes in chromosome 12 were imputed using the 1000 Genomes Phase 1 v3 reference panel. The reference datasets of all populations were downloaded from the IMPUTE2 website^35^. To minimise the computational intensity and increase efficiency, genotypes were pre-phased with SHAPEIT^36^ prior to imputation by IMPUTE2^35^ with the default options. As a post-imputation QC, SNPs were removed if MAF was low (<0.05) or the imputation info value was low (<0.8). As a result, the rs671 genotype in *ALDH2* became available in a total of 7,152 participants with expected MAF of 0.194 and imputation info value of 0.845.

### Statistical analysis

Statistical analyses were performed using Stata SE 12.0 (Stata Corp, Carollina, USA). First, the distribution of variable values was investigated. Fasting blood glucose, GGT and triglycerides were log-transformed to mimic a Gaussian distribution. No outliers were detected by visual inspection. Descriptive statistics of all variable values were presented as mean ± standard error for a continuous variable, and as number of counts and percentage for a categorical variable in men and women separately, according to their rs671 genotype in *ALDH2.* Apart from major dependent variables (e.g. hypertension) and major independent variables (e.g. alcohol intake), some variables included missing data points. Mean difference of these variables in men and women was evaluated through Student’s t-test for a continuous variable and by chi-squared test for a categorical variable. Similarly, mean differences in these variables among three different rs671 genotype groups were compared using one-way analysis of variance (ANOVA) for a continuous variable and using chi-squared test for a categorical variable; in men and women, and in male ever-drinkers and male never-drinkers, separately. In addition, in order to evaluate potential collider stratification bias, the difference of variable distribution by genotype between male ever-drinkers and male never-drinkers was tested using generalized regression models which include rs671 genotype, drinking behaviour history (never vs. ever) and interaction of genotype and drinking behaviour history as the independent variables.

The association between alcohol intake and other variables was assessed under an OLS regression model in men and women, separately. Continuous risk factors were predicted by alcohol intake under a linear regression model adjusting for potential confounding factors such as age, area, education, physical activity and smoking status. Hypertension, cardiovascular disease, coronary heart disease and diabetes were also predicted by alcohol intake under a logistic regression model adjusting for the same potential confounding factors. In order to investigate the potential violation of assumptions such as linearity of association and normality of the error distribution, plots of dependent variables against independent variables as well as plots of residuals against fitted values were generated. Results are presented as estimated regression coefficients β with 95% confidence interval (CI) for a continuous variable, and estimated odds ratio (OR) with 95% CI for a categorical variable. Corresponding p-values are also provided. The difference of estimates between men and women was assessed by Cochran’s Q test using fixed effect models assuming the true effect of alcohol is the same in men and women.

Lastly, the causal effect of alcohol intake on other variables was measured under an IV regression with a two stage least squares estimation method in men and women, separately, using the rs671 genotype as an instrument. For continuous risk factors, a two stage linear model was performed, with adjustments for age, area, education, physical activity and smoking status as well as additional adjustments for genotypic principal components to take into account population structure for the rs671 genotype. For hypertension and cardiovascular disease, a two stage logistic model was conducted; in the first stage, alcohol intake was predicted by rs671 genotype (with additive effect) under a linear regression model (adjusted for age, area, education, physical activity, smoking status and principal components); in the second stage, disease outcome was predicted by fitting the alcohol intake value from the first stage, under a logistic regression model (adjusted for the same potential confounding factors). Results were shown by providing estimated regression coefficients β with 95% CI for a continuous variable, and estimated OR, with 95% CI for a categorical variable along with corresponding p-values. The difference of estimates between men and women was assessed by Cochran’s Q test using fixed effect models assuming the true effect of alcohol is the same in men and women.

The same causal effect was then quantified in the whole population, using interaction of the rs671 genotype and sex as an instrument. Instrumental variable regression models were additionally adjusted for the rs671 genotype and sex, the variables that were used to compute the interaction. It should be noted that interaction of the rs671 genotype and sex was used as an instrument even if the rs671 genotype was directly included in the model^42^.

## Additional Information

**How to cite this article**: Cho, Y. *et al.* Alcohol intake and cardiovascular risk factors: A Mendelian randomisation study. *Sci. Rep.* **5**, 18422; doi: 10.1038/srep18422 (2015).

## Supplementary Material

Supplementary Information

## Figures and Tables

**Table 1:** Characteristics of study participants.

	All (N = 7,152‡)	Men (N = 3,365‡)	Women (N = 3,787‡)	P-value†
Lifestyle and socio-economic factor*				
Age (yrs)	52.3 ± 0.1	51.9 ± 0.2	52.7 ± 0.1	0.0002
Area (rural Ansung%/urban Ansan%)	47.2/52.8	43.1/56.9	50.8/49.2	<0.0001
Education (elementary school%/middle school%/high school%/university%)	33.2/22.9/30.7/13.2	19.7/22.4/36.2/21.6	45.1/23.3/25.9/5.7	<0.0001
Physical activity practitioner (%, N)	93.8 (6,609)	91.0 (3,027)	96.2 (3,582)	<0.0001
Ever smoker (%, N)	40.9 (2,884)	80.5 (2,698)	5.0 (186)	<0.0001
Current smoker (%, N)	22.2 (1,568)	44.2 (1,482)	2.3 (86)	<0.0001
Alcohol trait				
Ever drinker (%, N)	54.3 (3,849)	82.5 (2,763)	29.1 (1,086)	<0.0001
Current drinker (%, N)	47.8 (3,387)	72.4 (2,425)	25.7 (962)	<0.0001
Former drinker (%, N)	6.5 (462)	10.1 (732)	3.3 (124)	<0.0001
Never drinker (%, N)	45.7 (3,239)	17.5 (586)	71.0 (2,653)	<0.0001
Alcohol intake (g/day)	9.6 ± 0.3	18.8 ± 0.5	1.3 ± 0.1	<0.0001
Alcohol intake in current drinkers (g/day)	20.2 ± 0.5	26.1 ± 0.6	5.2 ± 0.3	<0.0001
γ-glutamyl transpeptidase (IU/L)	36.1 ± 0.8	55.4 ± 1.6	19.0 ± 0.3	<0.0001
Disease				
Hypertension (%, N)	39.4 (2,816)	41.7 (1,404)	37.3 (1,412)	<0.0001
Cardiovascular disease (%, N)	3.2 (228)	3.9 (130)	2.6 (98)	0.0020
Coronary heart disease (%, N)	1.7 (122)	2.1 (72)	1.3 (50)	0.008
Diabetes (%, N)	7.1 (504)	8.3 (278)	6.0 (226)	<0.0001
Cardiovascular risk factor				
Systolic blood pressure (mmHg)	124.9 ± 0.2	125.7 ± 0.3	124.1 ± 0.3	0.0004
Diastolic blood pressure (mmHg)	81.7 ± 0.1	83.4 ± 0.2	80.2 ± 0.2	<0.0001
Body mass index (kg/m^2^)	24.59 ± 0.04	24.24 ± 0.05	24.90 ± 0.05	<0.0001
Waist circumference (cm)	82.7 ± 0.1	83.7 ± 0.1	81.8 ± 0.2	<0.0001
Hip circumference (cm)	93.6 ± 0.1	93.6 ± 0.1	93.7 ± 0.1	0.6282
Waist to hip ratio (continuous)	0.883 ± 0.001	0.894 ± 0.001	0.873 ± 0.001	<0.0001
Fasting blood glucose (mg/dL)	87.7 ± 0.3	90.6 ± 0.4	85.0 ± 0.3	<0.0001
Total cholesterol (mg/dL)	191.9 ± 0.4	192.0 ± 0.6	191.8 ± 0.6	0.7511
HDL cholesterol (mg/dL)	44.7 ± 0.1	43.8 ± 0.2	45.5 ± 0.2	<0.0001
LDL cholesterol (mg/dL)	115.8 ± 0.4	114.5 ± 0.6	116.9 ± 0.5	0.0021
Triglycerides (mg/dL)	164.2 ± 1.3	179.9 ± 2.1	150.2 ± 1.5	<0.0001
Genotype				
rs671 in *ALDH2* (GG%/GA%/AA%)	71.0/26.2/2.7	71.2/26.0/2.9	70.9/26.5/2.6	0.7810
Population stratification indicator				
Height (cm)	160.0 ± 0.1	166.9 ± 0.1	153.8 ± 0.1	<0.0001
Principal component 1 (continuous)	0.0019 ± 0.0025	−0.0003 ± 0.0037	0.0038 ± 0.0034	0.4200
Principal component 2 (continuous)	0.0001 ± 0.0024	0.0001 ± 0.0036	0.0001 ± 0.0033	0.9956
Principal component 3 (continuous)	−0.0013 ± 0.0023	−0.0005 ± 0.0034	−0.0020 ± 0.0032	0.7419
Principal component 4 (continuous)	−0.0013 ± 0.0020	−0.0015 ± 0.0029	−0.0012 ± 0.0028	0.9382
Principal component 5 (continuous)	−0.0016 ± 0.0020	−0.0033 ± 0.0029	−0.0001 ± 0.0027	0.4252

*Values are represented as mean ± standard error for continuous variables and number of counts and percentage for categorical variables. ^†^P-values are from student’s t-test for continuous variables and chi-squared test for categorical variables assessing the difference between males and females. ^‡^Apart from major dependent variables (e.g. hypertension) and major independent variables (e.g. alcohol intake), some variables included missing data points.

**Table 2:** Characteristics of study participants according to their rs671 genotype in *ALDH2.*

	Men (N = 3,365‡)				Women (N = 3,787‡)			
	G/G (N = 2,395)	G/A (N = 874)	A/A (n = 96)	P-value†	G/G (N = 2,684)	G/A (N = 1,003)	A/A (N = 100)	P-value†
Lifestyle and socio-economic factor*								
Age (yrs)	51.8 ± 0.2	52.1 ± 0.3	51.6 ± 0.9	0.6015	52.6 ± 0.2	52.6 ± 0.3	53.3 ± 0.8	0.7561
Area (rural Ansung %/urban Ansan%)	43.8/56.2	40.7/59.3	45.8/54.2	0.2430	51.6/48.4	49.2/50.9	45.0/55.0	0.2030
Education (elementary school %/middle school %/high school %/university%)	20.5/21.9/36.1/21.5	17.7/23.1/37.1/22.1	19.0/29.5/30.5/21.1	0.3860	46.1/23.4/25.0/5.6	42.6/23.4/27.5/6.6	45.5/19.2/33.3/2.0	0.1150
Physical activity practioner (%, N)	91.6 (2,165)	90.0 (780)	87.2 (82)	0.1540	96.1 (2,538)	96.3 (947)	98.0 (97)	0.5950
Ever smoker (%, N)	80.7 (1,923)	80.9 (704)	74.0 (71)	0.2520	5.4 (142)	4.2 (41)	3.1 (3)	0.2110
Current smoker (%, N)	44.6 (1,062)	44.1 (384)	37.5 (36)	0.3940	2.4 (64)	2.0 (20)	2.0 (2)	0.7710
Alcohol trait								
Ever drinker (%, N)	93.0 (2,220)	60.7 (527)	16.8 (16)	<0.0001	35.6 (943)	14.3 (142)	1.0 (1)	<0.0001
Current drinker (%, N)	82.9 (1,979)	50.4 (437)	9.5 (9)	<0.0001	31.6 (837)	12.5 (124)	1.0 (1)	<0.0001
Former drinker (%, N)	10.1 (241)	10.4 (90)	7.4 (7)	0.6540	4.0 (106)	1.8 (18)	0 (0)	0.0010
Never drinker (%, N)	7.0 (166)	39.3 (341)	83.2 (79)	<0.0001	64.4 (1,705)	85.7 (849)	99.0 (99)	<0.0001
Alcohol intake (g/day)	23.78 ± 0.63	7.28 ± 0.59	0.41 ± 0.20	<0.0001	1.70 ± 0.13	0.41 ± 0.11	0.02 ± 0.02	<0.0001
Alcohol intake in current drinkers (g/day)	28.8 ± 0.7	14.6 ± 1.1	4.3 ± 1.7	<0.0001	5.4 ± 0.4	3.3 ± 0.8	2.3§	0.1186
γ-glutamyl transpeptidase (IU/L)	62.6 ± 2.1	38.6 ± 1.8	26.1 ± 2.0	<0.0001	19.5 ± 0.4	17.6 ± 0.5	17.6 ± 1.6	0.0095
Disease								
Hypertension (%, N)	43.8 (1,050)	36.8 (322)	33.3 (32)	<0.0001	37.8 (1,014)	36.2 (363)	35.0 (35)	0.6010
Cardiovascular disease (%, N)	3.6 (87)	4.6 (40)	3.1 (3)	0.4290	2.7 (71)	2.6 (26)	1.0 (1)	0.5960
Coronary heart disease (%, N)	2.0 (48)	2.6 (23)	1.0 (1)	0.4110	1.2 (33)	1.6 (16)	1.0 (1)	0.6610
Diabetes (%, N)	8.9 (212)	6.9 (60)	6.3 (6)	0.1460	6.0 (160)	5.9 (59)	7.0 (7)	0.9040
Cardiovascular risk factor								
Systolic blood pressure (mmHg)	126.5 ± 0.4	123.9 ± 0.6	123.2 ± 1.7	0.0003	124.1 ± 0.4	124.3 ± 0.6	123.8 ± 2.0	0.9553
Diastolic blood pressure (mmHg)	83.9 ± 0.2	82.3 ± 0.4	83.0 ± 1.1	0.0015	80.2 ± 0.2	80.2 ± 0.4	79.7 ± 1.1	0.9151
Body mass index (kg/m^2^)	24.3 ± 0.1	24.2 ± 0.1	23.8 ± 0.3	0.1949	24.9 ± 0.1	24.8 ± 0.1	24.9 ± 0.4	0.4150
Waist circumference (cm)	83.9 ± 0.2	83.2 ± 0.3	82.0 ± 0.7	0.0066	82.0 ± 0.2	81.3 ± 0.3	81.1 ± 1.0	0.1195
Hip circumference (cm)	93.6 ± 0.1	93.7 ± 0.2	93.0 ± 0.5	0.4840	93.8 ± 0.1	93.5 ± 0.2	93.3 ± 0.6	0.3782
Waist to hip ratio (continuous)	0.897 ± 0.001	0.888 ± 0.002	0.880 ± 0.005	<0.0001	0.874 ± 0.002	0.870 ± 0.003	0.869 ± 0.008	0.3304
Fasting blood glucose (mg/dL)	91.8 ± 0.5	88.3 ± 0.7	83.3 ± 1.3	<0.0001	84.9 ± 0.3	85.2 ± 0.7	86.7 ± 2.4	0.6904
Total cholesterol (mg/dL)	191.8 ± 0.8	192.9 ± 1.2	190.5 ± 3.7	0.6971	191.3 ± 0.7	192.9 ± 1.1	192.3 ± 3.7	0.4741
HDL cholesterol (mg/dL)	44.6 ± 0.2	41.8 ± 0.3	40.3 ± 0.8	<0.0001	45.6 ± 0.2	45.2 ± 0.3	46.1 ± 1.0	0.3771
LDL cholesterol (mg/dL)	112.4 ± 0.7	119.4 ± 1.1	120.4 ± 3.4	<0.0001	116.4 ± 0.6	118.2 ± 1.0	117.2 ± 3.0	0.3131
Triglycerides (mg/dL)	186.2 ± 2.6	166.0 ± 3.5	151.1 ± 7.9	<0.0001	149.0 ± 1.7	153.2 ± 3.1	150.6 ± 8.4	0.4184
Population stratification indicator								
Height (cm)	167.0 ± 0.1	166.8 ± 0.2	166.0 ± 0.6	0.2223	153.8 ± 0.1	153.8 ± 0.2	153.2 ± 0.5	0.5572
Principal component 1 (continuous)	0.017 ± 0.004	−0.005 ± 0.007	−0.006 ± 0.023	0.7174	0.003 ± 0.004	0.002 ± 0.007	0.046 ± 0.020	0.1273
Principal component 2 (continuous)	−0.006 ± 0.004	0.015 ± 0.007	0.021 ± 0.020	0.0203	0.002 ± 0.004	−0.003 ± 0.006	−0.016 ± 0.023	0.5925
Principal component 3 (continuous)	−0.002 ± 0.004	0.004 ± 0.007	−0.009 ± 0.023	0.6787	−0.001 ± 0.004	−0.005 ± 0.007	−0.006 ± 0.020	0.8225
Principal component 4 (continuous)	−0.003 ± 0.003	0.003 ± 0.006	−0.008 ± 0.017	0.6504	0.0001 ± 0.0033	−0.0083 ± 0.0054	0.0359 ± 0.0195	0.0389
Principal component 5 (continuous)	−0.001 ± 0.003	−0.007 ± 0.006	−0.018 ± 0.016	0.5092	0.0003 ± 0.0032	−0.0018 ± 0.0055	0.0056 ± 0.0166	0.8872

*Values are represented as mean ± standard error for continuous variables and number of counts and percentage for categorical variables. ^†^P-values are from ANOVA test for continuous variables and chi-squared test for categorical variables assessing the difference among G/G, G/A and A/A genotype groups. ^‡^Apart from major dependent variables (e.g. hypertension) and major independent variables (e.g. alcohol intake), some variables included missing data points. ^§^There is only one current drinker in the female A/A group.

**Table 3:** Characteristics of male participants according to drinking behaviour history and the rs671 genotype in *ALDH2.*

	Ever drinkers in men (N = 2,763††)					Never drinkers in men (N = 586††)					Men(N = 3,349††)
	G/G (N = 2,220)	G/A (N = 527)	A/A (N = 16)	P-value†	AdjustedP-value‡	G/G (N = 166)	G/A (N = 341)	A/A (N = 79)	P-value†	AdjustedP-value‡	Genotype*drinking behaviourhistory interactionp-value§
Lifestyle and socio-economic factor*											
Age (yrs)	51.7 ± 0.2	51.5 ± 0.4	51.2 ± 2.3	0.8668	0.548	52.1 ± 0.7	52.9 ± 0.5	51.7 ± 1.0	0.4405	0.974	0.978
Area (rural Ansung%/urban Ansan%)	43.9/56.1	40.4/59.6	31.3/68.8	0.2230	0.112	41.6/58.4	40.5/59.5	48.1/51.9	0.4630	0.904	0.475
Education (elementary school%/middle school%/high school%/university%)	20.6/22.0/36.4/20.9	16.9/20.5/37.8/24.7	25.0/25.0/25.0/25.0	0.2840	0.032	19.4/19.4/32.1/29.1	18.6/26.8/36.6/18.0	18.0/29.5/32.1/20.5	0.1070	0.615	0.147
Physical activity practitioner (%, N)	91.5 (2,016)	91.4 (480)	81.3 (13)	0.3440	0.532	92.5 (147)	87.9 (297)	88.3 (68)	0.2970	0.327	0.209
Ever smoker (%, N)	52.6 (1,167)	51.3 (270)	37.5 (6)	0.4260	—	15.1 (25)	42.4 (144)	43.0 (34)	<0.0001	—	<0.0001
Current smoker (%, N)	46.9 (1,039)	47.9 (252)	31.3 (5)	0.4100	—	13.3 (22)	38.2 (130)	39.2 (31)	<0.0001	—	<0.0001
Alcohol trait											
Alcohol intake (g/day)	25.6 ± 0.7	12.1 ± 0.9	2.4 ± 1.1	<0.0001	<0.0001	0	0	0	—	—	—
γ-glutamyl transpeptidase (IU/L)	65.0 ± 2.2	43.8 ± 2.6	28.3 ± 3.3	<0.0001	<0.0001	29.3 ± 2.0	30.8 ± 2.3	25.8 ± 2.4	0.6193	0.194	0.525
Disease											
Hypertension (%, N)	44.3 (984)	39.1 (206)	37.5 (6)	0.0830	0.018	36.1 (60)	33.1 (113)	31.7 (25)	0.7270	0.863	0.437
Cardiovascular disease (%, N)	3.6 (80)	4.8 (25)	0 (0)	0.3380	0.412	4.2 (7)	4.4 (15)	3.8 (3)	0.9710	0.735	0.925
Coronary heart disease (%, N)	2.1 (46)	3.0 (16)	0 (0)	0.3350	0.312	1.2 (2)	2.1 (7)	1.3 (1)	0.7470	0.919	0.806
Diabetes (%, N)	9.0 (199)	6.1 (32)	18.8 (3)	0.0340	0.125	7.8 (13)	7.9 (27)	3.8 (3)	0.4310	0.563	0.363
Cardiovascular risk factor											
Systolic blood pressure (mmHg)	126.6 ± 0.4	124.1 ± 0.7	121.5 ± 3.5	0.0091	0.001	124.5 ± 1.3	123.4 ± 0.9	123.3 ± 1.9	0.7471	0.904	0.537
Diastolic blood pressure (mmHg)	84.0 ± 0.2	83.2 ± 0.5	83.3 ± 2.1	0.3322	0.103	82.4 ± 0.9	80.9 ± 0.6	82.7 ± 1.2	0.1825	0.730	0.763
Body mass index (kg/m^2^)	24.3 ± 0.1	24.4 ± 0.1	24.3 ± 0.8	0.9063	0.796	24.3 ± 0.2	23.9 ± 0.2	23.6 ± 0.3	0.2405	0.269	0.087
Waist circumference (cm)	84.0 ± 0.2	83.8 ± 0.3	83.0 ± 2.1	0.8190	0.527	83.8 ± 0.6	82.3 ± 0.4	81.7 ± 0.8	0.0800	0.064	0.024
Hip circumference (cm)	93.6 ± 0.1	94.1 ± 0.2	95.4 ± 1.3	0.0793	0.045	93.7 ± 0.5	93.1 ± 0.3	92.5 ± 0.6	0.3337	0.347	0.131
Waist to hip ratio (continuous)	0.897 ± 0.001	0.891 ± 0.003	0.869 ± 0.015	0.0156	0.008	0.895 ± 0.005	0.883 ± 0.003	0.881 ± 0.006	0.1013	0.047	0.044
Fasting blood glucose (mg/dL)	91.9 ± 0.6	88.5 ± 0.8	84.9 ± 3.8	0.0081	0.001	91.5 ± 2.5	88.1 ± 1.1	83.1 ± 1.4	0.0267	0.044	0.011
Total cholesterol (mg/dL)	192.0 ± 0.8	194.0 ± 1.6	195.1 ± 11.0	0.5228	0.301	189.2 ± 2.9	191.4 ± 1.9	190.0 ± 4.0	0.7988	0.840	0.751
HDL cholesterol (mg/dL)	44.9 ± 0.2	42.6 ± 0.4	38.6 ± 1.9	<0.0001	<0.0001	40.6 ± 0.7	40.4 ± 0.5	40.8 ± 1.0	0.9372	0.628	0.995
LDL cholesterol (mg/dL)	112.1 ± 0.7	119.1 ± 1.5	121.5 ± 9.3	0.0001	<0.0001	117.7 ± 2.6	120.2 ± 1.7	120.6 ± 3.7	0.6746	0.526	0.455
Triglycerides (mg/dL)	187.7 ± 2.8	170.9 ± 4.7	178.9 ± 29.2	0.0093	0.003	164.7 ± 9.3	158.8 ± 5.1	145.5 ± 7.5	0.7895	0.410	0.660
Population stratification indicator											
Height (cm)	167.0 ± 0.1	167.1 ± 0.3	168.4 ± 1.4	0.6236	0.576	166.6 ± 0.5	166.4 ± 0.3	165.6 ± 0.6	0.4222	0.146	0.247
Principal component 1 (continuous)	0.0002 ± 0.0045	−0.012 ± 0.010	0.053 ± 0.056	0.3130	0.491	0.022 ± 0.016	0.007 ± 0.011	−0.016 ± 0.026	0.4140	0.198	0.198
Principal component 2 (continuous)	−0.006 ± 0.004	0.009 ± 0.009	0.029 ± 0.049	0.2839	0.128	−0.018 ± 0.017	0.024 ± 0.011	0.015 ± 0.022	0.1008	0.091	0.097
Principal component 3 (continuous)	−0.001 ± 0.004	−0.002 ± 0.008	−0.055 ± 0.056	0.5542	0.668	−0.023 ± 0.015	0.014 ± 0.011	0.003 ± 0.026	0.1446	0.223	0.164
Principal component 4 (continuous)	−0.003 ± 0.004	0.001 ± 0.007	−0.033 ± 0.041	0.7007	0.855	0.002 ± 0.014	0.005 ± 0.010	−0.007 ± 0.019	0.8614	0.425	0.806
Principal component 5 (continuous)	−0.002 ± 0.004	−0.004 ± 0.007	0.016 ± 0.029	0.9005	0.998	0.015 ± 0.013	−0.011 ± 0.009	−0.025 ± 0.018	0.1605	0.074	0.060

*Values are represented as mean ± standard error for continuous variables and number of counts percentage for categorical variables. ^†^P-values are from ANOVA test for continuous variables and chi-squared test for categorical variables assessing the difference among G/G, G/A and A/A genotype groups. ^‡^Adjusted p-values are from linear regression for continuous variables and logistic regression for categorical variables assessing the difference among G/G, G/A and A/A genotype groups, after adjustments for smoking behaviour (never/previous/current). ^§^P-values of interaction between genotype and drinking behaviour history (never vs. ever drinkers) are from generalized regression models. ^††^In men with drinking behaviour history available, some variables included missing data points apart from major dependent variables (e.g. hypertension) and major independent variables (e.g. alcohol intake).

**Table 4:** Ordinary least squares estimates of alcohol intake (g/day) to cardiovascular health outcomes.

	Men (N = 3,365‡)		Women (N = 3,787‡)		HeterogeneityP-value†
	OR (95% CI) by OLS estimation*	P-value	OR (95% CI) by OLS estimation*	P-value	
Disease					
Hypertension	1.007 (1.005, 1.010)	<0.0001	1.021 (1.008, 1.035)	0.002	0.048
Cardiovascular disease	0.995 (0.988, 1.003)	0.266	1.008 (0.976, 1.041)	0.618	0.937
Coronary heart disease	1.001 (0.992, 1.009)	0.868	1.018 (0.989, 1.047)	0.231	0.274
Diabetes	0.999 (0.994, 1.004)	0.756	1.002 (0.974, 1.030)	0.914	0.843
	Beta coefficient (95% CI) by OLS estimation*	**P-value**	Beta coefficient (95% CI) by OLS estimation*	**P-value**	
Cardiovascular risk factor					
Systolic blood pressure (mmHg)	0.075 (0.055, 0.095)	<0.0001	0.159 (0.061, 0.258)	0.002	0.099
Diastolic blood pressure (mmHg)	0.049 (0.035, 0.062)	<0.0001	0.154 (0.091, 0.216)	<0.0001	0.001
Body mass index (kg/m^2^)	0.004 (0.001, 0.008)	0.017	0.020 (0.003, 0.038)	0.024	0.104
Waist circumference (cm)	0.022 (0.012, 0.031)	<0.0001	0.046 (−0.001, 0.093)	0.054	0.328
Hip circumference (cm)	0.0057 (−0.0004, 0.0117)	0.068	0.0388 (0.0072, 0.0704)	0.016	0.043
Waist to hip ratio (continuous)	0.0002 (0.0001, 0.0002)	<0.0001	0.0002 (−0.0002, 0.0005)	0.386	0.869
Log-transformed fasting blood glucose (log(mg/dL))	0.0004 (0.0003, 0.0005)	<0.0001	0.0004 (0.0000, 0.0008)	0.043	<0.0001
Total cholesterol (mg/dL)	0.010 (−0.033, 0.052)	0.659	0.207 (0.017, 0.396)	0.033	0.048
HDL cholesterol (mg/dL)	0.059 (0.047, 0.071)	<0.0001	0.196 (0.142, 0.251)	<0.0001	<0.0001
LDL cholesterol (mg/dL)	−0.130 (−0.170, −0.090)	<0.0001	−0.023 (−0.191, 0.145)	0.786	0.226
Log-transformed triglycerides (log(mg/dL))	0.0010 (0.0008, 0.0013)	<0.0001	0.0005 (−0.0006, 0.0015)	0.393	0.327

*OR and beta coefficients by OLS estimation were obtained from standard regressions with an ordinary least squares estimation method (in logistic regression models and in linear regression models, respectively). All regression models were adjusted for age, area, education, physical activity and smoking status. ^†^Heterogeneity in estimates between males and females was assessed by Cochran’s Q test with fixed effects. ^‡^Apart from major dependent variables (e.g. hypertension) and major independent variables (e.g. alcohol intake), some variables included missing data points.

**Table 5:** Instrumental variable estimates of alcohol intake (g/day) to cardiovascular health outcomes, based on the rs671 genotype in ALDH2.

	Men(N = 3,365‡)		Women(N = 3,787‡)		Heterogeneity P-value†
	OR (95% CI) by IV estimation*	P-value	OR (95% CI) by IV estimation*	P-value	
Disease					
Hypertension	1.020 (1.010, 1.029)	<0.0001	1.042 (0.921, 1.178)	0.516	0.736
Cardiovascular disease	0.990 (0.968, 1.013)	0.390	1.206 (0.827, 1.759)	0.331	0.397
Coronary heart disease	0.994 (0.965, 1.024)	0.705	0.996 (0.614, 1.616)	0.988	0.994
Diabetes	1.018 (1.000, 1.036)	0.053	0.968 (0.768, 1.220)	0.783	0.662
	Beta coefficient (95% CI) by IV estimation*	**P-value**	Beta coefficient (95% CI) by IV estimation*	**P-value**	
Cardiovascular risk factor					
Systolic blood pressure (mmHg)	0.159 (0.085, 0.234)	<0.0001	−0.352 (−1.292, 0.589)	0.464	0.289
Diastolic blood pressure (mmHg)	0.085 (0.035, 0.135)	0.001	−0.109 (−0.701, 0.483)	0.718	0.522
Body mass index (kg/m^2^)	0.012 (−0.001, 0.025)	0.061	0.098 (−0.079, 0.274)	0.277	0.340
Waist circumference (cm)	0.060 (0.026, 0.094)	0.001	0.425 (−0.058, 0.909)	0.085	0.140
Hip circumference (cm)	0.005 (−0.017, 0.028)	0.633	0.358 (0.025, 0.690)	0.035	0.038
Waist to hip ratio (continuous)	0.0006 (0.0004, 0.0008)	<0.0001	0.0012 (−0.0023, 0.0047)	0.501	0.739
Log−transformed fasting blood glucose (log(mg/dL))	0.0010 (0.0006, 0.0014)	<0.0001	−0.0014 (−0.0052, 0.0025)	0.494	0.253
Total cholesterol (mg/dL)	−0.040 (−0.196, 0.116)	0.614	−0.897 (−2.817, 1.023)	0.360	0.383
HDL cholesterol (mg/dL)	0.170 (0.124, 0.216)	<0.0001	0.266 (−0.278, 0.810)	0.338	0.731
LDL cholesterol (mg/dL)	−0.405 (−0.552, −0.258)	<0.0001	−1.007 (−2.702, 0.688)	0.244	0.488
Log−transformed triglycerides (log(mg/dL))	0.002 (0.001, 0.003)	<0.0001	−0.007 (−0.018, 0.004)	0.222	0.139

*OR and beta coefficient by IV estimation were obtained from instrumental variable regressions with a two stage least squares estimation method (in logistic regression models and in linear regression models, respectively), using rs671 genotype as an instrument for alcohol intake. All regression models were adjusted for age, area, education, physical activity and smoking status. ^†^Heterogeneity in estimates between males and females was assessed by Cochran’s Q test with fixed effects. ^‡^Apart from major dependent variables (e.g. hypertension) and major independent variables (e.g. alcohol intake), some variables included missing data points.

**Table 6:** Instrumental variable estimates of alcohol intake (g/day) to cardiovascular disease and risk factors, based on interaction of the rs671 genotype in ALDH2 and sex.

	All (N = 7,152†)	
	OR (95% CI) by IV estimation*	P-value
Disease		
Hypertension	1.031 (1.001, 1.062)	0.040
Cardiovascular disease	0.949 (0.988, 1.004)	0.362
Coronary heart disease	0.984 (0.886, 1.093)	0.762
Diabetes	1.045 (0.989, 1.104)	0.117
	Beta coefficient (95% CI) by IV estimation*	**P-value**
Cardiovascular risk factor		
Systolic blood pressure (mmHg)	0.202 (0.087, 0.317)	0.001
Diastolic blood pressure (mmHg)	0.100 (0.025, 0.175)	0.009
Body mass index (kg/m^2^)	0.004 (−0.017, 0.024)	0.732
Waist circumference (cm)	0.023 (−0.032, 0.078)	0.410
Hip circumference (cm)	−0.025 (−0.061, 0.011)	0.179
Waist to hip ratio (continuous)	0.0005 (0.0001, 0.0009)	0.013
Log-transformed fasting blood glucose (log(mg/dL))	0.0012 (0.0006, 0.0017)	<0.0001
Total cholesterol (mg/dL)	0.038 (−0.197, 0.273)	0.750
HDL cholesterol (mg/dL)	0.166 (0.098, 0.233)	<0.0001
LDL cholesterol (mg/dL)	−0.362 (−0.578, −0.145)	0.001
Log-transformed triglycerides (log(mg/dL))	0.003 (0.002, 0.005)	<0.0001

*OR and beta coefficients by IV estimation were obtained from instrumental variable regressions with a two stage least squares estimation method (in logistic regression models and in linear regression models, respectively), using interaction of rs671 genotype and sex as an instrument for alcohol intake. All regression models were adjusted for age, area, education, physical activity and smoking status. ^†^Heterogeneity in estimates between males and females was assessed by Cochran’s Q test with fixed effects. ^‡^Apart from major dependent variables (e.g. hypertension) and major independent variables (e.g. alcohol intake), some variables included missing data points.
