# Corrigendum: Alcohol intake and cardiovascular risk factors: A Mendelian randomisation study

**DOI:** 10.1038/srep26307

**Published:** 2016-05-27

**Authors:** Yoonsu Cho, So-Youn Shin, Sungho Won, Caroline L Relton, George Davey Smith, Min-Jeong Shin

Scientific Reports 5: Article number: 1842210.1038/srep18422; published online: 12212015; updated: 05272016

This Article contains typographical errors in Table 6. In the column ‘OR (95% CI) by IV estimation’, the Cardiovascular disease OR value “0.949 (0.988,1.004)” should read: “0.949 (0.876, 1.029)”.

