# Supplementary material for: Alcohol intake and cardiovascular risk factors: A Mendelian randomisation study
Source: Sci Rep. 2015 Dec 21;5:18422. doi: 10.1038/srep18422 (PMC4685310; doi:10.1038/srep18422)
Supplement: Supplementary Information [file srep18422-s1.pdf]

< **Supplementary Information**>

Alcohol intake and cardiovascular risk factors: A Mendelian randomisation study

Yoonsu Cho, So-Youn Shin, Sungho Won, Caroline L Relton, George Davey Smith, Min-Jeong Shin

Supplementary figures and tables

Supplementary figure S1. Alcohol metabolism and related enzymes.

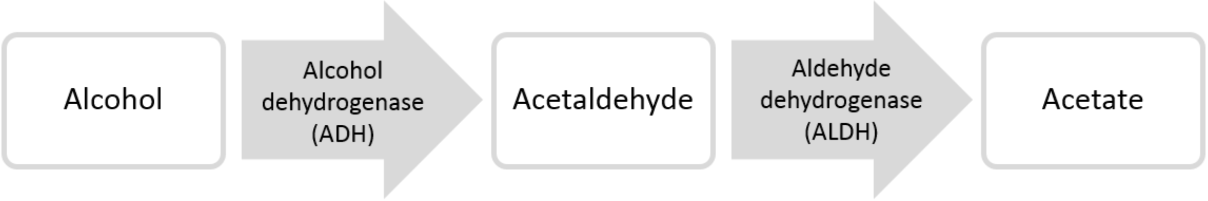

Supplementary figure S2. Distribution of rs671 ALDH2 genotype in the global scale. Image was generated in the Geography of Genetic Variants Browser (Beta v0.2)[1] with 1000 genomes (phase3, hg19) reference. Frequency scale is proportion out of 1 which is indicated by colours used in the pie chart.

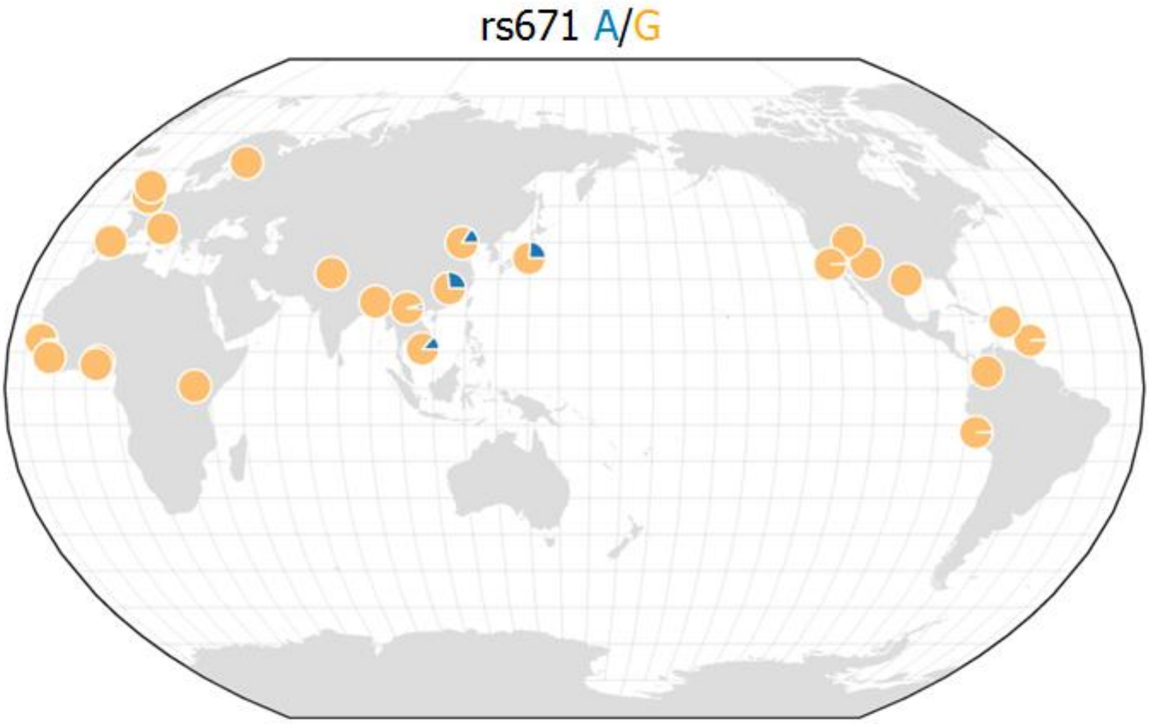

Supplementary Table S1. Ordinary least squares estimates of alcohol intake (g/day) to lifestyle and socio-economic factors

	Men (N=3,365[‡])		Women (N=3,787[‡])		Heterogeneity P-value[†]
	Beta coefficient (95% CI) by OLS estimation*	P-value	Beta coefficient (95% CI) by OLS estimation*	P-value	
Lifestyle and socio-economic factor					
Age (yrs)	-0.031 (-0.041, -0.020)	<0.0001	-0.191 (-0.239, -0.143)	<0.0001	<0.0001
Area (rural Ansung (ref))					
urban Ansan	0.00061 (0.00003, 0.00119)	0.040	0.006 (0.003, 0.008)	<0.0001	<0.0001
Education (elementary school (ref))					
middle school	-0.0001 (-0.0006, 0.0004)	0.745	0.0018 (-0.0005, 0.0041)	0.119	0.110
high school	0.0001 (-0.0005, 0.0007)	0.703	0.0027 (0.0029, 0.0050)	0.028	0.036
university	-0.0002 (-0.0007, 0.0003)	0.483	0.0002 (-0.0010, 0.0015)	0.706	0.560
Physical activity	0.0004 (0.0001, 0.0007)	0.018	0.0002 (-0.0008, 0.0012)	0.716	0.718
Current smoker	0.0024 (0.0018, 0.0029)	<0.0001	0.0024 (0.0016, 0.0032)	<0.0001	0.955

*Beta coefficients by OLS estimation were obtained from standard regressions with an ordinary least squares estimation method (in logistic regression models and in linear regression models, respectively). All regression models were adjusted for age, area, education, physical activity and smoking status.
[†]Heterogeneity in estimates between males and females was assessed by Cochran's Q test with fixed effects. [‡]Apart from major dependent variables (e.g. hypertension) and major independent variables (e.g. alcohol intake), some variables included missing data points.

Supplementary Table S2. Instrumental variable estimates of alcohol intake (g/day) to lifestyle and socio-economic factors, based on the rs671 genotype in ALDH2

	Men (N=3,365‡)		Women (N=3,787‡)		Heterogeneity P-value†
	Beta coefficient (95% CI) by IV estimation*	P-value	Beta coefficient (95% CI) by IV estimation*	P-value	
Lifestyle and socio-economic factor					
Age (yrs)	-0.013 (-0.052, 0.025)	0.489	-0.062 (-0.544, 0.420)	0.802	0.494
Area (rural Ansung (ref))					
urban Ansan	-0.001 (-0.003, 0.001)	0.309	-0.024 (-0.053, 0.004)	0.096	0.114
Education (elementary school (ref))					
middle school	-0.0014 (-0.0032, 0.0004)	0.129	0.0057 (-0.0170, 0.0284)	0.623	0.541
high school	0.0002 (-0.0019, 0.0023)	0.851	-0.0264 (-0.0516, -0.0011)	0.041	0.040
university	-0.0002 (-0.0020, 0.0016)	0.823	-0.0006 (-0.0131, 0.0119)	0.922	0.951
Physical activity	0.0012 (-0.0000, 0.0025)	0.057	-0.0043 (-0.0147, 0.0060)	0.412	0.301
Current smoker	0.0010 (-0.0011, 0.0032)	0.355	0.0028 (-0.0052, 0.0108)	0.489	0.669

*Beta coefficients by IV estimation were obtained from from instrumental variable regressions with a two stage least squares estimation method (in logistic regression models and in linear regression models, respectively), using rs671 genotype as an instrument for alcohol intake. All regression models were adjusted for age, area, education, physical activity and smoking status. †Heterogeneity in estimates between males and females was assessed by Cochran's Q test with fixed effects. ‡Apart from major dependent variables (e.g. hypertension) and major independent variables (e.g. alcohol intake), some variables included missing data points.

Supplementary Table S3. Ordinary least squares estimates of the rs671 genotype (major G allele as an effect allele, additive model) to lifestyle and socio-economic factors

	Men (N=3,365‡)		Women (N=3,787‡)		Heterogeneity P-value†
	Beta coefficient (95% CI) by OLS estimation*	P-value	Beta coefficient (95% CI) by OLS estimation*	P-value	
Lifestyle and socio-economic factor					
Age (yrs)	-0.200 (-0.768, 0.369)	0.491	-0.071 (-0.623, 0.482)	0.802	<0.001
Area (rural Ansung (ref))					
urban Ansan	-0.017 (-0.049, 0.015)	0.307	-0.028 (-0.058, 0.003)	0.078	<0.001
Education (elementary school (ref))					
middle school	-0.021 (-0.048, 0.006)	0.127	0.007 (-0.020, 0.033)	0.623	0.110
high school	0.003 (-0.028, 0.034)	0.851	-0.030 (-0.057, -0.003)	0.028	0.036
university	-0.003 (-0.030, 0.024)	0.824	-0.001 (-0.015, 0.014)	0.922	0.560
Physical activity	0.0181 (-0.0005, 0.0367)	0.056	-0.0050 (-0.0169, 0.0068)	0.407	0.718
Current smoker	0.015 (-0.017, 0.047)	0.359	0.003 (-0.006, 0.013)	0.492-	0.955

*Beta coefficients by OLS estimation were obtained from standard regressions with an ordinary least squares estimation method (in logistic regression models and in linear regression models, respectively). All regression models were adjusted for age, area, education, physical activity and smoking status.
†Heterogeneity in estimates between males and females was assessed by Cochran's Q test with fixed effects. ‡Apart from major dependent variables (e.g. hypertension) and major independent variables (e.g. alcohol intake), some variables included missing data points.

Supplementary Table S4. Ordinary least squares estimates of the rs671 genotype (major G allele as an effect allele, additive model) to cardiovascular disease and risk factors

	Men (N=3,365[‡])		Women (N=3,787[‡])		Heterogeneity P-value[†]
	OR (95% CI) by OLS estimation*	P-value	OR (95% CI) by OLS estimation*	P-value	
Disease					
Hypertension	1.332 (1.158, 1.532)	<0.0001	1.046 (0.909, 1.204)	0.529	0.705
Cardiovascular disease	0.873 (0.629, 1.212)	0.427	1.242 (0.804, 1.918)	0.328	0.237
Coronary heart disease	0.918 (0.591, 1.425)	0.702	0.994 (0.571, 1.729)	0.983	0.827
Diabetes	1.298 (1.000, 1.683)	0.050	0.963 (0.739, 1.255)	0.781	0.121
	Beta coefficient (95% CI) by OLS estimation*	P-value	Beta coefficient (95% CI) by OLS estimation*	P-value	
Cardiovascular risk factor					
Systolic blood pressure (mmHg)	2.357 (1.264, 3.451)	<0.0001	-0.420 (-1.530, 0.691)	0.459	<0.0001
Diastolic blood pressure (mmHg)	1.257 (0.520, 1.994)	0.001	-0.130 (-0.833, 0.573)	0.717	0.008
Body mass index (kg/m^2)	0.180 (-0.008, 0.367)	0.061	0.112 (-0.088, 0.313)	0.273	0.628
Waist circumference (cm)	0.882 (0.383, 1.381)	0.001	0.486 (-0.049, 1.021)	0.075	0.289
Hip circumference (cm)	0.081 (-0.252, 0.413)	0.634	0.408 (0.047, 0.769)	0.027	0.192
Waist to hip ratio	0.009 (0.005, 0.012)	<0.0001	0.001 (-0.003, 0.005)	0.500	0.003
Log-transformed fasting blood glucose (log(mg/dL))	0.015 (0.009, 0.020)	<0.0001	-0.002 (-0.006, 0.003)	0.490	<0.0001
Total cholesterol (mg/dL)	-0.594 (-2.907, 1.719)	0.614	-1.028 (-3.194, 1.138)	0.352	0.788
HDL cholesterol (mg/dL)	2.513 (1.858, 3.169)	<0.0001	0.305 (-0.323, 0.933)	0.341	<0.0001
LDL cholesterol (mg/dL)	-5.941 (-8.045, -3.837)	<0.0001	-1.171 (-3.111, 0.768)	0.236	0.001
Log-transformed triglycerides (log(mg/dL))	0.036 (0.021, 0.051)	<0.0001	-0.008 (1.830, 1.965)	0.2121.	<0.0001

*OR and beta coefficients by OLS estimation were obtained from standard regressions with an ordinary least squares estimation method (in logistic regression models and in linear regression models, respectively). All regression models were adjusted for age, area, education, physical activity and smoking status. [†]Heterogeneity in estimates between males and females was assessed by Cochran's Q test with fixed effects. [‡]Apart from major dependent variables (e.g. hypertension) and major independent variables (e.g. alcohol intake), some variables included missing data points.

References

1. Marcus, J. & Novembre, J. *Geography of genetic variants browser beta v0.2*. Available at: http://popgen.uchicago.edu/ggv.
